# Insulin Resistance and Endometrial Cancer: Emerging Role for microRNA

**DOI:** 10.3390/cancers12092559

**Published:** 2020-09-08

**Authors:** Iwona Sidorkiewicz, Maciej Jóźwik, Magdalena Niemira, Adam Krętowski

**Affiliations:** 1 Clinical Research Centre, Medical University of Białystok, M. Skłodowskiej-Curie 24a, 15-276 Białystok, Poland; magdalena.niemira@umb.edu.pl (M.N.); adamkretowski@wp.pl (A.K.); 2 Department of Gynecology and Gynecologic Oncology, Medical University of Białystok, M. Skłodowskiej-Curie 24a, 15-276 Białystok, Poland; jozwikmc@interia.pl; 3 Department of Endocrinology, Diabetology and Internal Medicine, Medical University of Białystok, M. Skłodowskiej-Curie 24a, 15-276 Białystok, Poland

**Keywords:** adipokines, endometrial cancer, estrogens, hyperinsulinemia, insulin, insulin resistance, insulin signaling, insulin-like growth factors, microRNA

## Abstract

**Simple Summary:**

Insulin resistance is one of the risk factors of endometrial cancer. Hyperinsulinemia can trigger many physiological effects that drive carcinogenesis, which is also modulated by epigenetic dysregulation including miRNAs expression. Our working hypothesis was that there must be a more pronounced relationship between insulin resistance and alterations in miRNA profiles of endometrial cancer patients. Consequently, this work was undertaken to better clarify this assumption. Our careful literature search indicated that miRNA could represent a potential molecular link between the metabolic alterations related to insulin resistance and endometrial cancer. Additionally, by reporting the known relationships between miRNA and both insulin resistance and endometrial cancer, we highlighted their potential role as predictive factors of future endometrial cancer in insulin resistant patients.

**Abstract:**

Endometrial cancer (EC) remains one of the most common cancers of the female reproductive system. Epidemiological and clinical data implicate insulin resistance (IR) and its accompanying hyperinsulinemia as key factors in the development of EC. MicroRNAs (miRNAs) are short molecules of non-coding endogenous RNA that function as post-transcriptional regulators. Accumulating evidence has shown that the miRNA expression pattern is also likely to be associated with EC risk factors. The aim of this work was the verification of the relationships between IR, EC, and miRNA, and, as based on the literature data, elucidation of miRNA’s potential utility for EC prevention in IR patients. The pathways affected in IR relate to the insulin receptors, insulin-like growth factors and their receptors, insulin-like growth factor binding proteins, sex hormone-binding globulin, and estrogens. Herein, we present and discuss arguments for miRNAs as a plausible molecular link between IR and EC development. Specifically, our careful literature search indicated that dysregulation of at least 13 miRNAs has been ascribed to both conditions. We conclude that there is a reasonable possibility for miRNAs to become a predictive factor of future EC in IR patients.

## 1. Introduction 

Endometrial cancer (EC) is the most common gynecological cancer in developed countries, with annual rates continuing to increase. It is estimated that more than 60,000 new EC diagnoses and 11,000 deaths from the disease occur in the United States alone every year [1]. However, the etiology of this disease is still not fully understood [1,2]. EC has been generally divided into two clinical categories. The first is classified as type I, which represents the vast majority (80–90%) of cases and is associated with a hyperplastic, low-grade, estrogen-related endometrium. It occurs primarily in obese pre-, peri-, and early postmenopausal women, and is associated with a good prognosis. Type II is characterized by a non-estrogenic, high-grade atrophic endometrium, which is also less well-differentiated. It occurs mostly in postmenopausal women and has a high risk of relapse and metastatic disease [3]. There are several histologic types of EC, and the most common endometrioid carcinoma tends to have a favorable prognosis. Other histotypes (such as serous or clear cell carcinoma) of EC are associated with a poor prognosis [4,5]. It was initially noted that type I EC generally presents an endometrioid morphology, whereas type II cancers are characterized by non-endometrioid histology, predominantly serous (Table 1). However, this classical distinction into two EC types has been challenged by long-term follow-up of patients with cancer of endometrioid histology and grades 2 and 3 of differentiation, whose survival turned out to be worse than expected [6,7]. In line with this, Setiawan et al. observed that the risk factor patterns of high-grade endometrioid tumors and type II tumors were similar [3]. Currently, only endometrioid grade 1 (well differentiated) EC is considered to be type I, with the remainder of EC cases being included into type II.

In 2013, The Cancer Genome Atlas (or TCGA) Research Network classification for EC applied four molecular subgroups: DNA polymerase epsilon (POLE)-mutated (ultra-mutated), microsatellite-instable (MSI-high, hypermutated), copy-number-low/protein 53 (P53)-wild-type (CNL), and copy-number-high/P53-mutant (CNH) [11]. The POLE and MSI groups suggest better prognosis of EC patients, and CNL and CNH groups are coupled to worse prognosis [13,14]. The implementation of this novel EC classification laid the grounds for the refined differential diagnosis of particular cancer subtypes based on molecular signatures and provided a precision approach for both research and clinical management [5]. A long-term follow-up of patients with these specific cancer subtypes is now mandatory and of utmost importance. 

Although EC is generally considered to be hormone-sensitive, its development is widely considered to also be regulated by environmental and lifestyle factors. One of this cancer’s risk factors is insulin resistance (IR), a prominent component in many metabolic disorders, including prediabetes, type 2 diabetes mellitus (T2DM), metabolic syndrome, and polycystic ovary syndrome (PCOS) [15,16,17,18]. IR is a condition of reduced sensitivity of insulin-responsive tissues to insulin, which leads to an increase in blood insulin and glucose concentrations. According to the International Diabetes Federation Diabetes Atlas, the global prevalence of T2DM developed from IR continues to be on the increase [19]. Hyperinsulinemia can trigger many physiological effects that drive carcinogenesis, as insulin is a major anabolic hormone that can stimulate cell proliferation [15]. Reduced receptor binding and decreased insulin receptor-mediated transduction lead to hyperinsulinemia which, in turn, triggers the deregulation of many metabolic pathways [20]. The exact molecular mechanisms linking IR and EC are still uncertain. However, the direct effect(s) on endometrial cells of insulin and insulin-like growth factors (IGFs), as well as of alterations in the mitogen-activated protein kinase (MAPK)/extracellular-signal-regulated kinase (ERK) and in the complex of phosphatidylinositol 3-kinase (P13K)–phosphatase and tensin homolog deleted on chromosome 10 (PTEN)–protein kinase B (Akt) signaling pathways, may play crucial roles [16,21,22]. 

Cancer development is also associated with epigenetic dysregulation, occurring at the earliest stage of cancer [23]. The most common epigenetic modifications are DNA methylation, histone methylation and acetylation, and the actions of non-coding RNAs, including microRNAs (miRNAs). All of them can regulate multiple genes and are involved in various important signaling pathways [24]. miRNAs belong to a class of highly conserved, sequence-specific, single-stranded, endogenous small non-coding RNAs, which bind to the 3’ end of the target mRNAs to induce their destabilization, degradation, and/or translation inhibition [25]. Deregulation of miRNA profiles has been implicated in a variety of cellular processes, including cancer development. Therefore, miRNAs have been drawing attention for their potential usefulness as diagnostic and/or prognostic markers [26,27].

To date, numerous studies have focused on the miRNAs’ role in endometrial carcinogenesis or IR, albeit no possible reciprocal interactions of miRNA and IR on EC have been taken into account [28]. Our working hypothesis was that there must be a more pronounced relationship between IR and alterations in miRNA profiles of EC patients. Consequently, this work was undertaken to better clarify this assumption. Furthermore, we discuss the known relationships between miRNA and both clinical conditions. 

## 2. Clinical Importance of the Association between Insulin Resistance and Dndometrial Cancer

Generally, IR is a principal pathophysiological process that relates not only to diabetes but also to prediabetes, as well as preclinical hyperinsulinemia and dysglycemia of varied degrees. IR has been defined as the resistance of target organs to the actions of insulin so that increased concentrations of this hormone are necessary to obtain a normal biological effect [29]. Accordingly, IR is the primary cause of T2DM and occurs years before its clinical manifestation [30]. This prediabetic state plays an important role in the development and progression of some types of cancers, including breast, prostate, colorectal, and endometrial neoplasia [31]. There is accumulating evidence that the risk factors for IR are also risk factors for EC, which strongly suggests that the development of IR and EC may be parallelly promoted at the same time. A meta-analysis conducted by Saed et al. demonstrated that diabetes increases the risk of EC by 72% [32]. Another work, a meta-analysis of 16 studies (3 cohort and 13 case-control studies), found that diabetes is associated with a 2.1-fold increase in the relative risk for EC [33]. Notably, a higher prevalence of EC was demonstrated in non-diabetic women with IR [34]. Decreased serum adiponectin (a polypeptide hormone increasing the cell’s insulin sensitivity and a surrogate marker for IR) concentration was found to be independently and inversely correlated with EC occurrence [35,36]. It has also been established that the EC risk increases quite shortly following the diagnosis of IR and diabetes; that is, approximately past 6 months after their detection [37]. Elevated levels of insulin in prediabetic and diabetic patients seem to affect their cancer risk rather quickly [38]. Similarly, epidemiological evidence shows that the presence of accompanying diseases substantially influences EC risk estimations [17]. For instance, the relationship between diabetes and EC incidence can be largely promoted by increased body weight [31]. In their pooled analysis of cohort studies, Stocks et al. found direct linear relationships of body mass index (BMI), blood pressure, blood glucose, triglycerides, and total cholesterol concentrations with EC risk [39]. This finding is particularly worrying in the present era of widespread overweight and obesity. 

## 3. Insulin Resistance as a Driving Force for Endometrial Cancer

**Figure 1:**
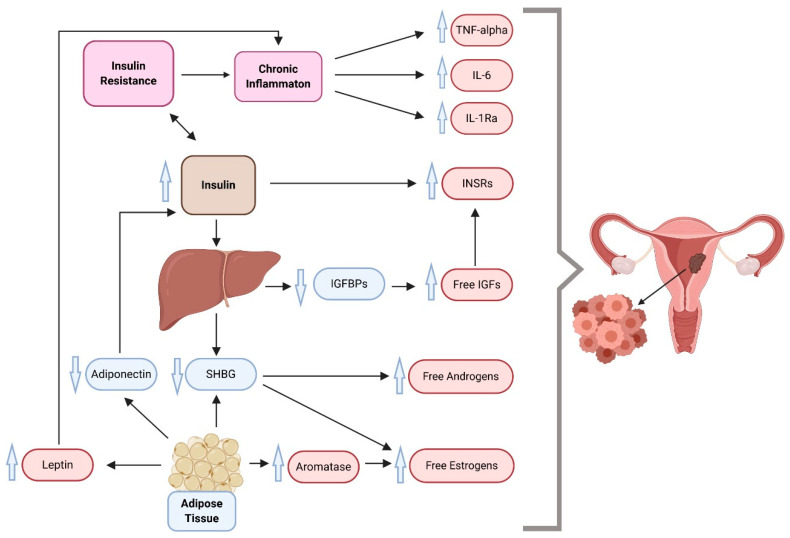
A proposed multidimensional model of endometrial cancer development, which suggests insulin resistance, inflammation, and overweight/obesity as driving forces behind cancer. IGFs, insulin-like growth factors; IGFBPs, insulin-like growth factor binding proteins; IL-1Ra, interleukin 1 receptor antagonist; IL-6, interleukin 6; INSR, insulin receptor; SHBG, sex hormone-binding globulin; TNF-alpha, tumor necrosis factor-alpha. Compiled from References [35,43,44,45,46,47].

Over past decades, hyperinsulinemia and IR have been implicated as playing a major role in diabetes-promoted cancers. Multiple studies were able to demonstrate a direct association between IR and the incidence of EC with several biological mechanisms as a result of their common regulation by molecular factors (such as mediators of inflammation and adipokines) [40,41,42]. Figure 1 presents a model of links between metabolic alterations in the development of this malignancy, highlighting the roles of changes in the insulin and IGF system and mediators of inflammation. 

### 3.1. Insulin Receptor

Molecular signaling downstream of insulin receptor (INSR) is tightly regulated by a large number of factors. This control system supervises energy homeostasis in peripheral target tissues for insulin. Both insulin and IGF1 activate a specific tyrosine kinase and the two main pathways of insulin signaling are the PI3K/Akt and the MAPK/ERK pathways. These two metabolic cascades contain several points of regulation, crosstalk with other signaling pathways, and control proliferation, differentiation, and survival at the cellular level [48,49]. 

Insulin signal transduction occurs through two INSR isoforms resulting from transcriptional alternative splicing: INSR-A and INSR-B, differing by the absence (INSR-A) or presence (INSR-B) of exon 11. INSR-A is the principal receptor during fetal development, recognizes both insulin and IGFs, demonstrates a greater affinity for IGF2 than IGF1, and is responsible for intracellular signaling that results in mitogenic responses. INSR-B, expressed in mature insulin-sensitive tissues, is quite insulin-specific and primarily involved in glucose homeostasis [50]. The differences in the effects exerted by INSR-A and INSR-B could be due to their varying abilities to bind IGF2 [51]. INSR-A overexpression was found in many cancer cells and tissues, suggesting that INSR-A-mediated signaling pathways may contribute to cancer pathogenesis [52]. Wang et al. demonstrated that the total *INSR* and *INSR-A* mRNA levels and the ratio of *INSR-A*/total *INSR* were significantly higher in EC than in control endometrium [53]. However, no comparisons between clinical types I and II of EC, or between particular EC histotypes, were made, making the interpretation of these results somewhat difficult. On the contrary, Flannery et al. found that *INSR-B* expression was increased in non-diabetic patients both in complex endometrial hyperplasia and EC, relative to normal tissue [54].

### 3.2. Insulin-Like Growth Factors and Their Receptors 

Combined, insulin, IGF1, and IGF2 critically control many aspects of metabolism, growth, and survival. IGFs are predominantly produced in the liver by hepatocytes in response to growth hormone (GH) stimulation [55]. IGF1 displays significant amino acid sequence homology with insulin and enhances insulin sensitivity [56]. To date, IGF2 actions have been insufficiently characterized; however, some relevant roles have been determined for fetal development and cerebral protection [51]. Both ligands IGF1 and IGF2 activate the IGF1 receptor (IGF1R), making it their common receptor. The IGF system plays a central role in human carcinogenesis. Interestingly, it has been hypothesized that IGF2 is more closely linked to the etiology of EC than IGF1 [57]. 

At the pathophysiological level, insulin can also bind to IGF1R, which is a cell-surface tyrosine kinase receptor coupled to several intracellular secondary messenger pathways, including the PI3K/Akt signaling cascade. IGF1R plays a pivotal role in cell survival by regulating somatic growth, development, and metabolism, as demonstrated by using *IGF1R* knock-out mice that displayed severe growth deficiency, lethal neonatal lung hypoplasia, and muscle hypoplasia [58]. Although INSR and IGF1R are highly homologous and are coupled to similar intracellular pathways, insulin and IGFs stimulate distinct and specific functions, such as glucose metabolism for insulin and cell growth and proliferation for IGFs [59]. Additionally, the functional specificity of insulin/INSR signaling can be affected by: (1) crosstalk between INSR-A and IGF1 because of the abundant synthesis of INSR-A in tissues and its increased binding affinity for IGF1, (2) enhanced formation of an INSR-A/IGF1R hybrid receptor, and (3) autocrine and/or paracrine IGF production [60]. There is convincing evidence for a direct effect of insulin and IGF1 on EC cells, with the activation of the INSR resulting in both increased cell proliferation and inhibition of apoptosis [55]. From Dai et al., although serum concentrations of IGF1 and IGF2, as well as the degree of activation of IGF1R in endometrial cells did not differ between diabetic patients with or without EC, both the degree of activation of IGF2R and of PI3K were significantly higher in endometrial cells in T2DM patients with EC [61]. These same authors suggested that increased IGF2R protein expression in endometrial cells in T2DM patients could increase PI3K/cyclin D1 (CCND1)-dependent cell growth through the loss of competitive binding of IGF2 to IGF1R, a possible explanation for the higher risk of developing EC in T2DM. Moreover, this study indicated that IGF1 and IGF2 compete for binding to IGF1R, whereas binding of IGF2 to IGF1R may cause alternative phosphorylation of IGF1R with the resultant suppression of downstream PI3K and CCND1 signaling cascades [61]. Somewhat similar conclusions were obtained by Petridou et al., who suggested a more pronounced role of IGF2 than IGF1 in the etiology of EC [57]. Of clinical relevance, Price et al. noted that increased IGF1R expression is linked to higher BMI and better overall survival and disease-free survival in EC [62]. Gunter et al. observed that free IGF1 levels were inversely associated with the incidence of EC [63]. Of interest, Roy et al. suggested the existence of two different mechanisms that activate IGF1R in EC: ligand-dependent in type I and ligand-independent in type II [64]. These authors found that *IGF1* mRNA expression was increased in type I compared with type II.

Further interesting findings were reported by Ding et al., who found that higher protein expression of IGF1, IGF1R, and INSR in colorectal cancer was associated with a history of diabetes, suggesting that IGF1/INSR signaling may play an important role in the development of this cancer in diabetic patients [65]. Unfortunately, many clinical trials with anti-IGF1R showed only limited responses in small proportions of cancer patients. Despite promising preclinical data, anti-IGF1R/INSR-targeted therapies lacked overall efficacy and the multitude of side effects led to their discontinuation [50,66,67]. In contrast, metformin (an oral antidiabetic drug of the biguanide family used for the treatment of T2DM) is known to interact with the IGF pathway, induces apoptosis, and inhibits proliferation and migration of EC cells [68]. Three separate systematic reviews and meta-analyses confirmed a beneficial role of metformin in improving overall survival and progression-free survival in EC [69,70,71]. In an in vitro study, metformin even inhibited proliferation and migration of endometrial serous carcinoma cell lines. The authors suggested that this drug could be a novel and attractive therapeutic approach for the treatment of this highly aggressive variant of EC [68].

### 3.3. Insulin-Like Growth Factor Binding Proteins

Although IGF1 is structurally related to insulin, unlike insulin, it circulates in the blood bound to specific carrier proteins, called IGF binding proteins (IGFBPs), with variable affinity. IGFBPs tightly regulate IGF1 availability by increasing its half-life, usually by forming a tertiary complex that blocks IGF1 from binding to IGF1R. Six IGFBPs (labeled IGFBP-1 to -6) have been identified so far [72]. Hyperinsulinemia has been shown to increase hepatic production and bioavailability of IGF1, in part by inhibiting hepatic production of IGFBP-1 and -2. This surplus IGF1 may excessively activate IGF1R, INSR/IGF1R, and proliferative and anti-apoptotic signaling in both premalignant and malignant tissues [45]. Insulin-sensitizing, blood pressure-lowering, and antiatherosclerotic properties of IGFBP-1 have been demonstrated, raising the possibility that increasing IGFBP-1 levels may be a therapeutic option to protect individuals from IR, arterial hypertension, and atherosclerosis [73]. However, high IGFBP-1 concentrations seem to be associated with EC risk in older women and women with an elevated BMI [74]. One study, by Weiderpass et al., found an increased risk for EC based on serum levels of IGFBP-1 solely in women who had ever used hormonal replacement therapy [75].

Recent evidence suggests beneficial effects of IGFBP-2 on systemic metabolism by indirect interacting with IGF1 signaling, including inhibition of adipogenesis and enhanced long-term insulin sensitivity [76,77]. Besides binding to IGFs, IGFBP-2 interacts with cellular components and exerts other key functions within the nucleus, directly or indirectly promoting transcriptional activation of specific genes. IGFBP-2 activities, both IGF-dependent and IGF-independent, contribute to the protein’s functional roles in growth, development, metabolism, and malignancy [78]. The overexpression of IGFBP-2 has been shown to correlate with tumor progression in a number of cancers, including ovarian, lung, and pancreatic cancer [79,80,81,82]. However, the mechanisms by which IGFBP-2 contributes to the progression of cancer are still unclear [83].

As for IGFBP-3, this protein inhibits adipocyte differentiation and impacts the peroxisome proliferator-activated receptor-gamma (PPARγ) system, suggesting a role for IGFBP-3 in the pathogenesis of obesity and IR. Apart from physiological IGF-dependent effects, this carrier protein has been demonstrated to regulate cell proliferation independently of binding to IGFs [84]. Mochizuki et al. found that the anti-proliferative and proapoptotic activities of IGFBP-3 are IGF-independent and attenuate epidermal growth factor (EGF)-induced EC cell proliferation. However, the exact details of action by which IGFBP-3 inhibits the EGF-mediated survival pathway require elucidation [85]. Recently, the binding of IGFBP-3 to a variety of growth factors was shown to improve the efficacy of anti-cancer precision therapy, counteract numerous mechanisms of tumor resistance, and combat tumor heterogeneity [86].

### 3.4. Estrogens

Estrogens are important participants in the metabolic regulation, playing a mitogenic role in the normal endometrium [87]. The effects of estrogen are mostly mediated by three receptors: two cytosolic estrogen receptors (ER), α (ERα) and β (ERβ), and transmembrane G protein-coupled estrogen receptor 1 (or GPER). ERs can carry out both genomic (transcription and gene expression regulation) and nongenomic (regulatory protein modifications) signal transduction. Estradiol (E2), the principal biologically active form of estrogen, controls insulin activity directly via actions on insulin-sensitive tissues or indirectly by regulating factors responsible for oxidative stress, with both outcomes contributing to IR [88]. Of note, some data suggest opposed effects of ERα and ERβ on glucose tolerance, and that ERβ ligands exert diabetogenic actions [89].

Estrogen signaling causes proliferation of EC [90]. Extensive crosstalk between estrogen signaling and the insulin/IGF axis was recently thoroughly discussed [91]. Research on ER-positive breast cancer (cell line MCF-7 xenografts) demonstrated that tamoxifen, a selective ER modulator, effectively inhibits classical ER-dependent transcription, including the transcription of *IGF1R* gene product [92]. In vivo studies have shown that E2 improves insulin sensitivity and glucose tolerance via activation of ERα/PI3K/Akt signaling [93,94]. However, E2 has been demonstrated to inhibit the in vitro binding of insulin to INSR by binding to both insulin and its receptor instead, an observation strongly suggesting the ability of E2 to induce IR either directly or indirectly [95]. Attention should be paid to tissue-specific roles of E2 [96]. Estrogens mediate the expression of IGFs in the uterus, but IGFBPs also interfere with this process [55,97]. Merritt et al. observed lower expression of *IGF1*, but higher expression of *IGFBP-1* and *IGFBP-3*, coupled with higher protein expression for ER, INSR, and IGF1R in postmenopausal endometrium as compared to premenopausal proliferative phase endometrium [98].

Several EC risk factors provide strong support for the hypothesis of the causative role of unopposed estrogen, stating that EC risks are increased in women with high plasma estrogens and/or low progesterone, so that estrogenic effects are not sufficiently counterbalanced by the latter [99,100,101]. Insulin and IGF1 have been shown to stimulate ovarian steroid synthesis, resulting in cellular proliferation and inhibition of apoptosis in breast epithelium and endometrium [102]. Estrogens, both internal and from external provision, play a significant role in EC development. The majority of type I EC cases express ER, and higher ER expression has been associated with better clinical outcomes [8]. A recent analysis by Tian et al. underlined that insulin and estrogens could exert combined or even synergistic effects on the progression of type I EC [103]. Estrogens are thought to trigger proliferation and growth in cancer cells through the activation of ERα and the subsequent activation of PI3K and MAPK pathways [90]. Furthermore, an in vitro study showed that insulin directly stimulates aromatase activity in both endometrial glands and stroma, which strongly suggests that hyperinsulinemia caused by IR predisposes to EC by enhancing endogenous endometrial estrogen production [104]. Work by Galvão Wolff et al. indicated that IR stimulates endometrial expression of ERs and progesterone receptors (PRs), thereby contributing to the increased occurrence of endometrial proliferative lesions [105].

### 3.5. Sex Hormone-Binding Globulin

The activity of estrogens depends on their bioavailability, which is primarily determined by sex hormone-binding globulin (SHBG). The synthesis of SHBG occurs in the liver and is increased by sex steroids (mostly estrogens) and thyroxine, whereas insulin is a known important inhibitor of its production. Winters et al. found lower serum SHBG and lower hepatic *SHBG* expression with increasing IR, together with a weak association between fasting insulin concentration and *SHBG* mRNA expression [106]. Not only insulin but also IGF1 inhibits the hepatic production of SHBG [102].

In premenopausal women, hyperinsulinemia promotes the stimulation of ovarian androgen synthesis and decreases hepatic production of SHBG. This leads to increased circulating levels of free androgens. In contrast, in postmenopausal women, adipose tissue is the main source of estrogens through the aromatization of androgens. Obesity and hyperinsulinemia, if present, further lead to increased aromatization of androgens and decreased production of SHBG, the results of which are increased levels of bioavailable estrogen [107]. Excessive estrogens promote the development of EC, as described by the unopposed estrogen hypothesis.

### 3.6. Adipokines

The adipose tissue secretes a wide variety of bioactive molecules, including adipokines and hormones, such as adiponectin, leptin, resistin, visfatin, and chemerin, as well as proinflammatory cytokines, such as tumor necrosis factor-α (TNF-α) [108]. Among adipokines, adiponectin proved to be particularly important. Decreased adiponectin production exerts a key role in the pathogenesis of obesity-associated disorders: arterial hypertension, metabolic syndrome, atherosclerosis, and cancer [109,110,111]. Two separate studies demonstrated a significant relationship between high circulating adiponectin levels and reduced EC risk, an observation largely independent of other obesity-related risk factors [112,113].

Leptin has contrasting biological functions to adiponectin: it decreases tissue sensitivity to insulin and increases plasma insulin concentration. Hyperinsulinemia and obesity are therefore linked with high leptin and low adiponectin levels [114]. The role of leptin in IR is still not fully clarified, but solid evidence indicates that leptin is a major metabolic regulator of circulating IGFBP-2 [115,116]. A relationship between serum leptin and insulin concentrations has been confirmed, regardless of body fatness [117,118,119]. Since obesity and adipokines are independent risk factors for EC, this notion supports the roles of two distinct mechanisms involved in endometrial carcinogenesis: excess estrogen and IR [120,121]. Resistance to leptin is considered a hallmark of obesity and has been shown to lead to hepatic IR [122]. Importantly, leptin plays a proinflammatory role, contributing to the generation and maintenance of low-grade inflammation, recently also linked to EC [42]. However, literature data regarding circulating levels of adiponectin and leptin in EC report conflicting results [111,123,124]. Increased circulating adiponectin and adiponectin/leptin ratio and decreased leptin concentration were shown to be associated with reduced risk for EC [125]. On the other hand, Ma et al. observed increased leptin and decreased adiponectin levels in EC [126]. Unfortunately, adiponectin and leptin concentrations and insulin pathway receptor expressions were not found useful for defining molecular subtypes of EC [127]. Moreover, molecular links between adipokines and cancer cells are complex and as yet, are not fully understood [128]. It has been hypothesized that circulating levels of adiponectin and leptin, together with insulin pathway molecules, exert oncogenic effects on endometrial tissue not only through their impact on the expression of tumor cell receptors but also by the activation of multiple epigenetic pathways within neoplastic cells and their microenvironment [129,130].

Other adipokines/cytokines of adipose origin such as visfatin, vaspin, and omentin display proinflammatory properties and affect insulin sensitivity and secretion. Recent research indicates that serum visfatin concentration is elevated in patients with EC and that combined serum visfatin and resistin levels could be used to predict the risk of advanced stages of EC [131]. Hlavna et al. showed increased circulating levels of resistin in EC patients compared with control subjects [132]. Unraveling the pathophysiological roles of adipokines in IR and EC should be prioritized in future research [133].

## 4. miRNAs in Both Insulin Resistance and Endometrial Cancer

Post-transcriptional regulation by miRNAs is of interest as a mechanism to silence gene expression [134]. Aberrant expression of the miRNA profile plays a key role in a wide variety of physiological processes, including cell proliferation, apoptosis, and tissue differentiation [135]. Yet, deregulation in miRNA biogenesis and function have been shown to modulate many fundamental signaling pathways, including insulin synthesis, secretion, and signal transduction, and therefore, specific miRNA patterns are likely to play a role in the development of IR and related metabolic complications [136]. Importantly, miRNA-mediated insulin signaling modulation is tissue- and cell-specific, with distinct miRNAs modulating components of the insulin transduction pathway only in some tissues or cells. The basis for IR is multifactorial and includes obesity, inflammation, mitochondrial dysfunction, endoplasmic reticulum stress, oxidative stress, lipotoxicity/hyperlipidemia, genetic background, and hypoxia. These factors contribute quite differently to the disruption of insulin signaling [137].

Various conditions are caused by dysregulation of gene networks due to changes in miRNA expression, and the association between miRNAs and cancer is currently under vivid investigation [138]. miRNAs regulate cell metabolic processes either directly by targeting key molecules of metabolic pathways (transporters and enzymes, including kinases), or indirectly by modulating the expression of important transcription factors [139].

On the one hand, EC molecular subtypes have been shown to demonstrate distinct miRNA signatures. These miRNA signatures are reduced, and particular levels of depletion are characteristic for particular EC subtypes [140]. In summary, many miRNAs, either circulating or of tissue origin, have been found dysregulated in EC. Table 2 presents their comprehensive list.

On the other hand, at least several miRNAs are known to be involved in the pathogenesis of cancer. As for endometrial neoplasia, a 4-miRNA signature (miR-4758, miR-876, miR-142, miR-190b) has been established as an independent prognostic factor for overall survival in EC patients (area under the curve (AUC) of receiver operating characteristic (ROC) curve was 0.7 at 5-year overall survival) [150]. By contrast, based on their systematic review, Donkers et al. proposed miR-205, the whole miR-200 family, miR-135b, miR-182, miR-183, and miR-223 as promising diagnostic biomarkers in EC [151]. Such studies were performed in the hope that the expression pattern of miRNA would become an early diagnostic and prognostic biomarker, whilst particular miRNAs could be identified as novel therapeutic targets.

Although the pathophysiology that underlies the association of IR with EC requires further investigation, miRNAs may be a missing link. Of interest, our careful literature search indicated that dysregulation of at least 13 miRNAs is actually shared by or has been ascribed to both IR and EC. Table 3 substantiates these findings.

The let-7 miRNA family, whose decreased expression in EC tissues has been demonstrated, is also involved in the development of IR [179]. A let-7 loss contributes to carcinogenesis via an increase in its target oncogenes (such as high-mobility group AT-hook 2 (*HMGA2)*, *c-Myc*, Janus protein tyrosine kinase (*JAK*)*,* Aurora B kinase, and signal transducer and activator of transcription 3 (*STAT3*)) and stemness factors [153,154,180]. However, the overexpression of let-7 in mouse skeletal muscles is related to the impairment of glucose tolerance and enhancement of IR [181]. The lin-28/let-7 axis regulates the insulin/PI3K/mammalian target of rapamycin (*mTOR*) pathway via multiple targets, such as *IGF1R*, *INSR*, and insulin receptor substrates 1 and 2 (*IRS-1*, *IRS-2*), thereby directly regulating glucose metabolism [152,182]. Understanding the tissue-specific regulation of let-7 may fill the current data gap and result in its potential use as a therapeutic for an array of metabolic diseases [183].

Increased expression of miR-9 in EC versus normal endometrial tissue has been shown [184,185,186]. Myatt et al. demonstrated that miR-9 was increased in EC tissue but lower in HEC-1B (type II EC cell line) compared with Ishikawa cells (type I EC line). Moreover, miR-9 expression was inversely correlated with forkhead transcription factor 1 (*FOXO1*) expression both in EC in vivo and in Ishikawa cells [155]. Two studies reported that the gene for sirtuin 1 (*SIRT1*), together with *FOXO1* and the gene for sterol regulatory element-binding protein 1 (*SREBP-1*) act as a pathway involved in tumorigenesis suppression and play a role in the development of progestin resistance in EC cells [187,188]. Since *FOXO1* and *SREBP-1* are targets of insulin, their role in IR can be hypothesized. miR-9 can regulate insulin secretion by inhibiting transcription factor onecut-2 (*OC-2*) and *SIRT1* in vivo in pancreatic β-islets [156]. In turn, this decrease in *OC-2* in insulin-secreting cells results in an increase in the expression of its target gene, granuphilin, a key player in insulin secretion and known to negatively regulate insulin exocytosis [189]. mir-9 expression was first thought to be restricted to the brain and pancreatic islets, yet recent studies emphasize the need to focus on its precise functional role in cancer [190].

Members of the miR-29 family (i.e., miR-29a, b, and c) have been shown to be involved in the EC development [191]. Specifically, miR-29b was found to play important roles in proliferation and progression in EC cells by direct regulation of *PTEN*, whose involvement in inhibiting cell migration, invasion, and cytoskeleton rearrangement has been proven [161]. Chen et al. demonstrated that miR-29b contributes to EC angiogenesis by targeting both MAPK/ERK and PI3K/Akt signaling pathways [160]. Significant downregulation of miR-29c was observed to occur in EC, possibly resulting in increased cell proliferation and collagen type IV alpha 1 synthesis [163]. The miR-29 family is involved in IR, as its in vivo suppression in adult mice led to a significant reduction of fasting blood glucose concentration and improvement in insulin sensitivity [192]. A study by Massart et al. reported that miR-29a and miR-29c expression are increased in skeletal muscle from patients with T2DM, playing a pivotal role in glucose and fatty acid metabolism [162]. In line with this, silencing miR-29a resulted in decreased glucose transport and altered lipid metabolism in myotube cells, indicating the involvement of this miRNA in IR by targeting peroxisome proliferator-activated receptor δ (*PPARδ*) in skeletal muscle [157]. The various mechanisms of action by the miR-29 family suggest its dichotomous role as a tumor suppressor and oncogene based on tissue specificity [193,194].

Other miRNAs involved in both IR and EC are miR-103 and miR-107. An in vitro study by Du et al. showed that miR-103 overexpression significantly promoted EC cell proliferation, whereas downregulation significantly suppressed EC cell proliferation [195]. miR-103 has been demonstrated to directly target tissue inhibitor of metalloproteinase 3, leading to an imbalance between matrix metalloproteinases and their tissue inhibitors, well known to play a critical role in tumor development [164]. Hepatic miR-103 overexpression in obese mice promotes glucose intolerance and IR [165]. In turn, miR-107-5p promotes EC progression and invasion by targeting *ERα* [166]. miR-103/107 inactivation leads to increased expression of caveolin-1 (*CAV1*) in adipocytes, thereby reducing downstream insulin signaling and decreasing adipocyte size [165]. Interestingly, miR-103 and miR-107 target RNase III-like enzyme named DICER, which is a key component of the miRNA processing machinery, resulting in global miRNA inhibition. However, these inhibiting effects may also be mediated by other miRNAs [140].

miR-126 has been reported to directly target *IRS-1* in SK-Hep1 hepatocytes [167]. This miRNA was found to be frequently downregulated in EC. Moreover, *IRS-1* is involved in miR-126-mediated EC cell migration and invasion, thus raising a possibility of miR-126-based molecular targeted therapy for EC [168].

High expression of the miRNA-200 family (including miR-141, miR-200a, miR-200b, miR-200c, and miR-429) has been demonstrated in endometrioid EC compared with normal endometrium, suggestive of their substantial role in cancer growth [196]. Importantly, miR-200 has been implicated in IR by inducing pancreatic β-cell proinflammatory state and damage, and by downmodulating *IRS-2* [197,198]. An in vitro study by Lu et al. showed that the expression of the miR-200 family in Ishikawa cells (type I EC cell line) was increased when compared with HEC-1B cells (type II EC line). There is convincing evidence that dynamic expression changes during transition from the normal to cancerous state reflect a link between ovarian steroids and the miRNA expression pattern [199]. Zinc finger E-box binding homeobox 1 (*ZEB1*) is a target gene of miR-200. The product of this gene is involved in epithelial–mesenchymal transition (EMT), which contributes to cancer invasion, metastasis, recurrence, and therapeutic resistance [200,201,202]. Therefore, there should be a role for miR-200 in EMT. Upregulation of miR-141 has been demonstrated in IR, as well as in EC [169,170,196]. The increased expression of miR-141 resulted in impaired glucose-stimulated insulin secretion and pancreatic β-cell proliferation. In addition, a positive correlation was observed in diabetic patients between miR-141 expression and blood glucose concentration. Forkhead box A2 (*FOXA2*) was identified as a direct miR-141 target gene [170]. Separate work demonstrated that *FOXA2* must be important in tumorigenesis based on its role in the inhibition of EMT in cancer [203,204].

Interestingly, miR-221 and miR-222 are also related to both IR and EC. Ramon et al. showed a significant downregulation of miR-221/222 in endometrioid EC in comparison with control endometrium. miR-221 and -222 were negatively correlated with the vascular endothelial growth factor A (VEGF-A) protein level, an observation suggesting their involvement in the mechanism of increased VEGF-A ratios observed in EC. miR-222-3p expression was found lower in ERα-positive EC tissues as compared with ERα-negative ones [205]. Consequently, the level of miR-222-3p expression was lower in tumors of lower grades and earlier stages. Further, regulation of *ERα* expression by miR-222-3p was confirmed in RL95-2 EC cells [176]. Overexpression of miR-221 caused inflammation and IR in differentiated 3T3-L1 adipocytes through the suppression of *SIRT1* [173]. An in vitro study on preadipocytes demonstrated that leptin and TNF-α downregulate miR-221, which inversely affects the adiponectin receptor 1 (*ADIPOR1*) and transcription factor v-ets erythroblastosis virus E26 oncogene homolog 1 (*ETS1*) expression. Adiponectin signaling promotes insulin sensitivity, and *ETS1* is known to regulate the expression of cytokines, chemokines, and other genes involved in angiogenesis [206]. The association of miR-221 in the complex interplay between *ERα, PR*, hypoxia-inducible factor 1-alpha (*HIF1-α*), and zinc finger protein SNAI2 (snail family transcriptional repressor 2) (*SLUG*) has been demonstrated and ascribed to EMT in endometrioid EC [174].

A recent communication reported on downregulated expression of miR-320a in EC [177]. From Abbas et al., miR-320a induces proliferation inhibition in EC cells, *IGF1R* is a direct functional mediator for miR-320a, and IGF1R is a critical negative regulator of insulin sensitivity in the endothelium [207]. Additionally, a study by Ling et al. showed that miR-320 increases insulin sensitivity of insulin-resistant 3T3-L1 adipocytes [178]. miR-320 may inhibit insulin/PI3K signaling in adipocytes, leading to IR; thus, anti-miR-320 oligo has been proposed as a potentially new therapeutic strategy to control IR [178].

Another aspect of the EC relationship with IR is the pleiotropic function of adipokines. Adipokine-regulated miRNAs can act as either oncogenic or anti-tumoral factors [208]. Moreover, adipose tissue is a major source of circulating miRNAs and they constitute a novel class of adipokines that can act as regulators of metabolism in tissues other than fat [209,210]. The discovery of cell signaling mechanisms followed by the appreciation of a wide network of miRNA-target genes’ expression patterns has been crucial to identify the adipokine-regulated miRNAs in the development of EC. However, the data from human patients are limited and large in vivo studies are needed [211].

It has been hypothesized that the role of miRNAs in the metabolic crosstalk is not only between cellular and non-cellular components within the tumor microenvironment but also between cancerous and other cells, such as adipocytes [174]. Another suggestion is that transfer of specific adipose cell- or other cell-derived miRNAs may be involved in the regulation of endometrial tumor progression, providing a new form of intercellular communication. Overexpressed miRNAs are included in exosomes released from cells and play a functional role in cell-to-cell communication [212].

## 5. Perspective and Future Directions

Although diabetes has long been known to be an independent risk factor for EC, little is known about the relationships between IR and EC [213]. Literature data were unable to indicate clear associations between insulin, IGFs, and sex steroid hormones with EC incidence because of the multitude of dysregulated pathways that lead to EC progression. Yet, meta-analyses support the theory about the association between IR and EC. Currently, there is still a need for new precise molecular tools for the early diagnosis, risk assessment, and prediction of EC development, and miRNA may be a promising marker [214].

Epidemiological and cohort studies should determine the risk of EC in patients with IR based on miRNA expression pattern. That would allow timely intervention(s) to prevent cancer development. Patients with T2DM, prediabetes, metabolic syndrome, and PCOS should be included.

Taking into account that EC is a hormone-dependent cancer, studies on epigenetic mechanisms, including miRNA and sex steroid pathway profiling, both in cancer and IR, are worth undertaking. EC molecular subtypes have been shown to demonstrate distinct miRNA signatures [141]. These miRNA signatures are reduced, and particular levels of depletion are characteristic for particular EC subtypes [140]. A long-term follow-up of patients with these specific cancer subtypes is now mandatory to unveil the clinical significance of miRNA signatures. Similarly, long-term studies should reveal the significance of miRNAs in reference to type I and II EC. The thirteen miRNAs found by us to be dysregulated in both IR and EC are worth special attention.

## 6. Conclusions

This review highlights changes in miRNA involved in both IR and EC. In support of the possible role of miRNA in both conditions, our careful literature search found that dysregulation of at least 13 miRNAs has been ascribed to both IR and EC. Therefore, miRNA could represent a potential molecular link between the metabolic alterations related to IR and EC. There is a reasonable possibility for miRNAs to become a predictive factor of future EC in IR patients.

## Figures and Tables

**Table 1:** Conventional comparison between type I and type II endometrial cancer [8,9,10,11,12].

Characteristic Feature	Type I	Type II
Frequency	~80% of cases	Up to 20% of cases
Estrogenic status	Estrogen-dependent	Estrogen-independent
Histology	Mostly endometrioid adenocarcinomas	Non-endometrioid carcinoma
Precursor lesion	Atypical hyperplasia	Endometrial intraepithelial carcinoma
Growth	Slow growth	Rapid growth
Risk factors	Imbalance between estrogen and progesterone exposures (such as the use of unopposed estrogen therapy)	Early age at menarche, low parity, tobacco smoking
Obesity	Often present	Often absent
Type 2 diabetes mellitus	Often present	Often absent
Estrogen and progesterone receptors	Usually ER (+), PR (+)	Usually ER (−), PR (−)
Prognosis	Usually good prognosis	Poor prognosis
*PTEN* Mutations	Yes	No
P53 Overexpression	No	Yes
Other Frequent Mutations	*ARID1A* *PIK3CA* *CTNNB1* *FGFR2*	*PPP2R1A* *FBXW7* *HER2*

*ARID1A*: AT-Rich Interaction Domain 1A; *CTNNB1*: Catenin Beta 1; ER: Estrogen Receptor; *FBXW7*: F-Box and WD Repeat Domain-containing 7; *FGFR2*: Fibroblast Growth Factor Receptor 2; *HER2*: Human Epidermal Growth Factor Receptor 2; *PIK3CA*: Phosphatidylinositol-4,5-Bisphosphate 3-Kinase Catalytic Subunit Alpha; *PPP2R1A*: Protein Phosphatase 2 Scaffold Subunit A alpha; PR: Progesterone Receptor; *PTEN*: Phosphatase and tensin homolog deleted on chromosome 10; P53: Protein 53.

**Table 2:** MicroRNAs (miRNAs) found as dysregulated in endometrial cancer (EC).

miRNA	Form of Dysregulation	Studied Specimen	Reference Specimen	Reference
hsa-miR-1307-3p; hsa-miR-183-3p; hsa-miR-183-5p; hsa-miR-200b-3p; hsa-miR-429	up	EC	Normal endometrium	[141]
hsa-miR-152-3p; hsa-miR-24-1-5p; hsa-miR-374b-5p; hsa-miR-542-3p	down	EC	Normal endometrium	[141]
miR-650; miR-168; miR-572; miR-200a; miR-182; miR-622; miR-34a; miR-205	up	Endometrioid EC	Benign endometrium	[142]
miR-411; miR-487b	down	Endometrioid EC	Benign endometrium	[142]
let-7c-5p; miR-125b-5p; miR-23b-3p; miR-99a-5p	down	Endometrioid EC	Non-neoplastic endometrium	[143]
let-7g-5p; miR-195-5p; miR-34a-5p; miR-497-5p	down	Endometrioid EC (grade 1+2) and serous EC	Non-neoplastic endometrium	[143]
miR-205; miR-182; miR-325; miR-183; miR-203; miR-210; miR-223; miR-194; miR-95; miR-151; miR-200a; miR-301; miR-141; miR-215; miR-103; miR-106a; miR-191; miR-184; miR-326; miR-34a; miR-200c; miR-23a	up	Endometrioid EC	Normal endometrium	[144]
miR-1; miR-101; miR-10b*; miR-127–3p; miR-132*; miR-133a; miR-133b; miR-136; miR-136*; miR-139-5p; miR-140-3p; miR-140-5p; miR-142-3p; miR-142-5p; miR-143; miR-143*; miR-145; miR-145*; miR-152; miR-195; miR-196b; miR-199a-5p; miR-199b-3p; miR-199b-5p; miR-214; miR-214*; miR-23b; miR-24-1*; miR-27b; miR-299-3p; miR-299-5p; miR-29b; miR-33a; miR-337-5p; miR-34b; miR-34b*; miR-34c-5p; miR-376a; miR-376c; miR-377; miR-379; miR-381; miR-410; miR-411; miR-424; miR-450a; miR-455-3p; miR-455-5p; miR-497; miR-503; miR-542-3p; miR-542-5p; miR-654-3p; miR-873	down	Serous EC	Normal endometrium	[145]
miR-222; miR-223; miR-186; miR-204	up	Serum of endometrioid EC patients	Serum of healthy controls	[146]
miR-186; miR-222; miR-223	up	Serum of EC patients	Serum of healthy controls	[147]
miR-204	down	Serum of EC patients	Serum of healthy controls	[147]
let-7g*; miR-181c*; miR-516a-3p; miR-9; miR-203; miR-375; miR-652; miR-146a; miR-9*; miR-210; miR-32; miR-148a; miR-425; miR-592; miR-21; miR-7-1*; miR-107	up	Endometrioid EC	Normal endometrium	[148]
miR-502-3p; miR-130a; miR-214; miR-218; miR-99a; miR-410; miR-100; miR-199a-3p; miR-424; miR-199a-5p; miR-214*; miR-99a*; let-7c; miR-212, miR-130a*; miR-495; miR-100*; miR-125b*; miR-218-2*; miR-502-5p; miR-532-5p	down	Endometrioid EC	Normal endometrium	[148]
miR-31; miR-995-5p; miR-490-3p; miR-644; miR-522; miR-519d; miR-98; miR-425; miR-518e; miR-155	up	Serous EC	Normal endometrium	[148]
miR-370; miR-423-5p	down	Serous EC	Normal endometrium	[148]
miR-516; let-7a; miR-424; miR-496; miR-409; miR-451; miR-431; miR-516; miR-503; miR-369; miR-032; miR-032b; miR-425; miR-181c; miR-19b; miR-009; miR-205; miR-423; miR-223; miR-183; miR-146; miR-200c	up	Endometrioid EC	Normal endometrium	[149]

Nomenclature was given as provided by cited references. EC: endometrial cancer. *: the less expressed strand.

**Table 3:** miRNAs found as dysregulated in both insulin resistance (IR) and EC.

miRNA	Form of Dysregulation	Target Genes Involved in IR	Target Genes Involved in EC	Reference
let-7	down	*IGF1R, IGF2BP-2, INSR, IRS-1, IRS-2*	*HMGA2, c-myc, JAK,* Aurora B kinase, *STAT3*	[152,153,154]
miR-9	up	*OC-2, SIRT1*	*FOXO1*	[155,156]
miR-29a	up	*PPARδ*	*TPX2*	[157,158]
miR-29b	up	*CAV2, INSIG1, PIK3R1*	*PTEN*	[159,160,161]
miR-29c	down	*HK2, GLUT1, IRS-1*	*COL4A1*	[162,163]
miR-103	up	*CAV1*	*TIMP-3*	[164,165]
miR-107	up	*CAV1*	*ERα*	[165,166]
miR-126	down	*IRS-1*	*IRS-1*	[167,168]
miR-141	up	*FOXA2*	*PLA2R*	[169,170]
miR-200	up	*ZEB1*	*PTEN*	[171,172]
miR-221	up	*SIRT1*	*SLUG*	[173,174]
miR-222	up	*IRS-1*	*ERα*	[175,176]
miR-320a	up	*PI3Kp85*	*IGR1R*	[177,178]

*CAV1*, caveolin-1; *CAV2*, caveolin-2; *ERα*, estrogen receptor alpha; *FOXA2*, forkhead box A2; *FOXO1*, forkhead transcription factor 1; *GLUT1*, glucose transporter 1; *HK2*, hexokinase 2; *HMGA2*, high mobility group AT-hook 2; *IGFBP-2*, insulin-like growth factor binding protein 2; *IGF1R*, insulin-like growth factor receptor; *INSIG1*, Insulin-Induced Gene 1; *INSR*, insulin receptor; *IRS-1*, Insulin receptor substrate 1; *IRS-2*, Insulin receptor substrate 2; *JAK*, Janus protein tyrosine kinase; *OC-2*, Transcription Factor Onecut-2; *PI3Kp85*, phosphoinositide 3-kinase regulatory subunit p85; *PIK3R1*, phosphatidylinositol 3-kinase regulatory subunit; *PLA2R*, phospholipase A2 receptor; *PTEN*, phosphatase and tensin homolog deleted on chromosome 10; *SIRT1*, Sirtuin 1; *SLUG*, Zinc Finger Protein SNAI2 (Snail Family Transcriptional Repressor 2); *STAT3*, signal transducer and activator of transcription 3; *TIMP-3*, tissue inhibitor of metalloproteinase 3; *ZEB1*, zinc finger E-box-binding homeobox 1.
